# From Setback to Success: A Case Report of Secondary Surgical Strategies for Liposclerosing Myxofibrous Tumor‐Related Fractures

**DOI:** 10.1002/ccr3.73234

**Published:** 2026-07-29

**Authors:** Yingsong Yuan, Yang Gu

**Affiliations:** ^1^ Department of Radiology Ningbo No. 6 Hospital Ningbo Zhejiang China; ^2^ Ningbo Clinical Research Center for Orthopedics Sports Medicine & Rehabilitation Ningbo Zhejiang China; ^3^ Department of Trauma Orthopedic Surgery Ningbo No. 6 Hospital Ningbo Zhejiang China

**Keywords:** bone grafting, internal fixation, liposclerosing myxofibrous tumor, pathological fracture, secondary surgery

## Abstract

This case highlights that an early pathological fracture may occur after curettage and synthetic bone grafting for a distal femoral liposclerosing myxofibrous tumor despite prophylactic fixation. Prompt revision with graft debridement, autologous iliac crest grafting, and stable distal femoral plate fixation can achieve fracture union, pain‐free function, and excellent recovery.

## 1 Introduction

Liposclerosing myxofibrous tumor (LSMFT), first described by Ragsdale and Sweet in 1986 [1], is a rare benign bone tumor characterized by a heterogeneous histological composition of mature adipose tissue, fibrous stroma, myxoid matrix, and scattered calcifications [2, 3]. The tumor typically affects the metaphyses of long bones, with the distal femur and proximal tibia being the most common sites [4, 5]. Clinically, LSMFT often presents as an asymptomatic lesion incidentally detected on imaging, although some patients may experience localized pain or swelling. In rare cases, pathological fractures may occur as the initial manifestation due to tumor‐induced bone weakening [3, 5].

Primary treatment for LSMFT usually involves curettage and bone grafting (autologous, allogeneic, or synthetic) for symptomatic or large lesions to prevent pathological fractures [6]. While the recurrence rate after primary resection is relatively low (approximately 5%–10%) [3], pathological fractures at the previous resection site represent a rare but clinically significant complication. To date, only a handful of cases have been reported in the literature, and consensus on the optimal secondary treatment approach remains lacking [7].

In this report, we present a pathological fracture that occurred 25 days after initial curettage, synthetic bone grafting, and prophylactic plate fixation for LSMFT of the distal femur. We describe the clinical presentation, radiological findings, revision surgical management, and follow‐up outcomes. This case report follows the CARE guidelines to support complete and transparent reporting.

## 2 Case Presentation/Examination

A 38‐year‐old man was initially evaluated at a local hospital in November 2024 for a 3‐month history of intermittent right knee pain. Physical examination showed localized tenderness over the right distal femur without swelling or joint effusion. Following the primary procedure described below, he sustained a minor fall 25 days after surgery and presented to the emergency department with acute right knee pain and inability to bear weight. Examination revealed marked knee swelling, ecchymosis over the distal femur, and severe tenderness at the previous surgical site. Knee range of motion was limited to 0°–20°, and longitudinal percussion tenderness was present. Distal pulses and sensation were intact.

## 3 Differential Diagnosis, Investigations and Treatment

**FIGURE 1:**
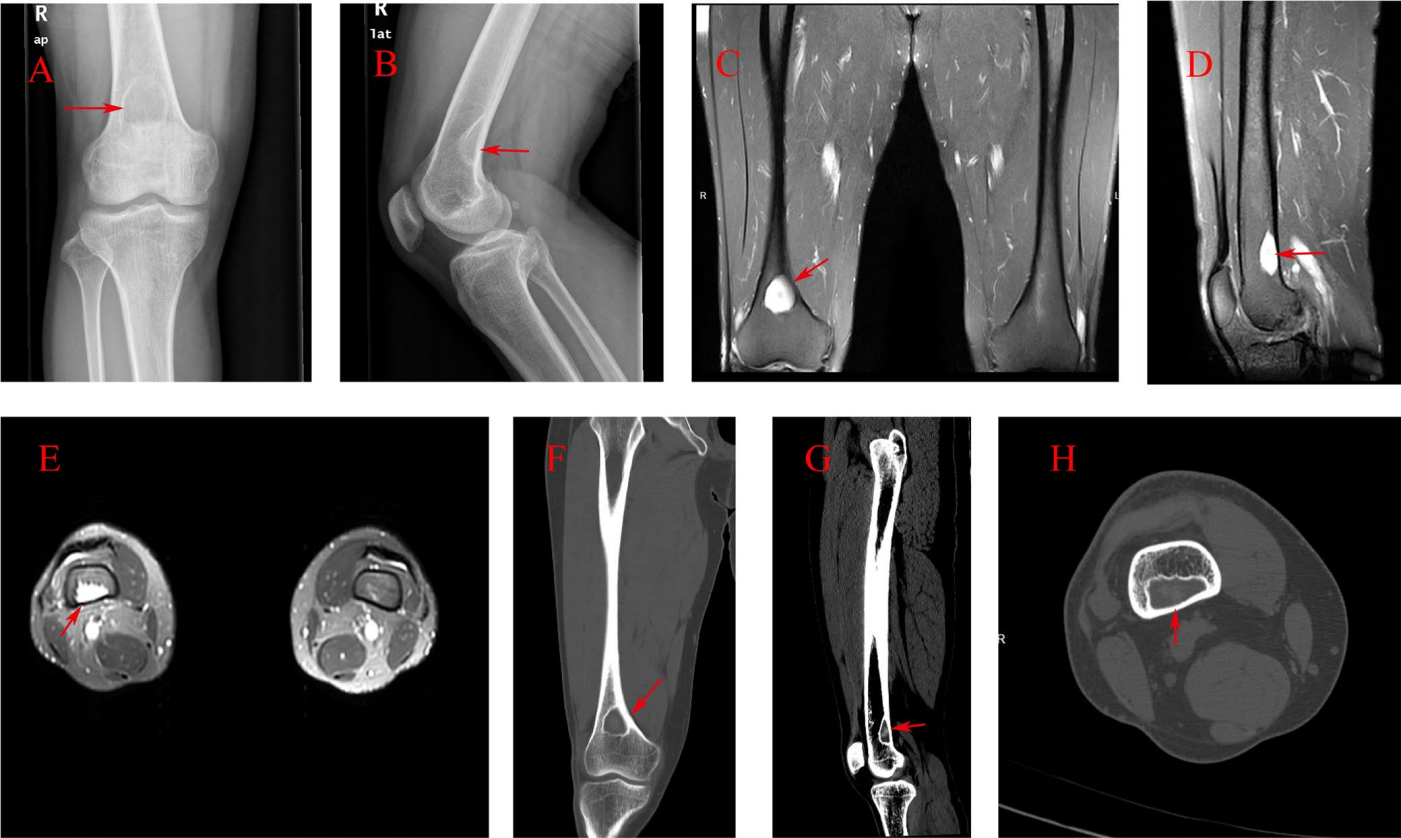
Imaging findings of the initial liposclerosing myxofibrous tumor (LSMFT) in the right distal femur. (A,B) Anteroposterior and lateral radiographs showing a benign‐appearing cystic lesion in the distal femoral metaphysis (red arrows). (C–E) Magnetic resonance images (C, coronal T2‐weighted; D, sagittal T2‐weighted; E, axial T2‐weighted) showing a cystic lesion at the distal femur (red arrows). (F–H) Computed tomography images (F, coronal; G, sagittal; H, axial) showing a 1.4 cm × 2.8 cm × 3.2 cm benign‐appearing lesion of suspected fibrous origin (red arrows).

Initial radiographs demonstrated a 1 cm × 3 cm × 3 cm benign‐appearing cystic lesion in the metaphysis of the right distal femur (Figure 1A,B). Magnetic resonance imaging in February 2025 showed a cystic lesion at the distal femur that was initially considered a benign bone cyst (Figure 1C–E). Computed tomography in April 2025 demonstrated a 1.4 cm × 2.8 cm × 3.2 cm benign‐appearing metaphyseal lesion of suspected fibrous origin (Figure 1F–H). The imaging differential diagnosis included a simple bone cyst, fibrous dysplasia, an intraosseous lipoma, and LSMFT; histopathological examination was required for definitive diagnosis.

**FIGURE 2:**
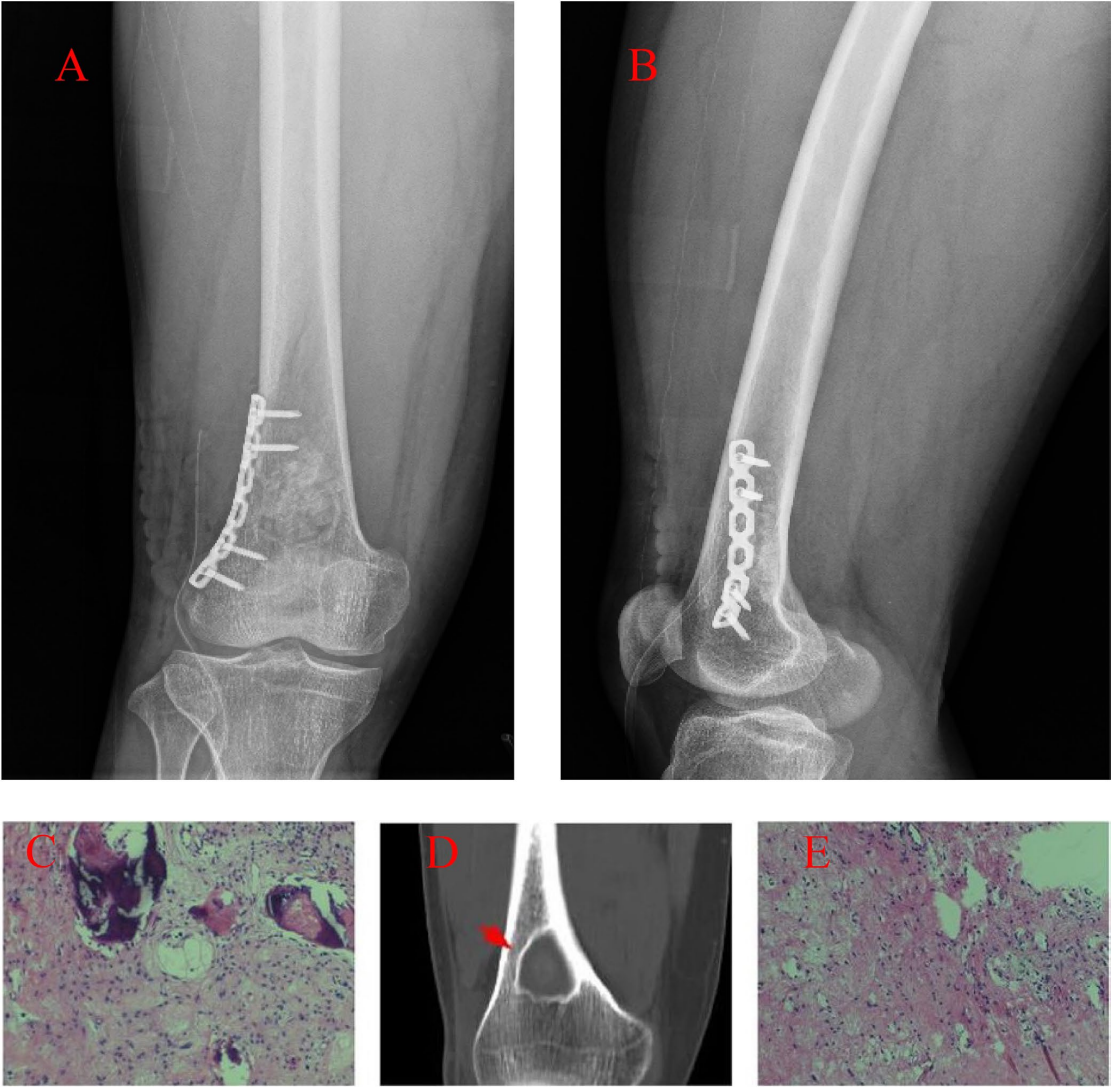
Initial surgical intervention and histopathological findings. (A, B) Anteroposterior and lateral radiographs after curettage, bone grafting, and prophylactic fixation with a 6‐hole locking compression plate. (C, E) Hematoxylin and eosin‐stained sections showing the heterogeneous fibro‐osseous features of LSMFT, including fibrous and myxoid stroma with cystic change (C, original magnification ×10; E, original magnification ×10). (D) Coronal computed tomography image of the lesion (red arrow).

The patient underwent curettage and excision of the lesion, followed by filling of the defect with a calcium phosphate bone graft substitute (Rebone, China). A 6‐hole locking compression plate and screws (LCP; Mindray, China) were used for prophylactic reinforcement (Figure 2A,B). Histopathological examination showed a benign fibro‐osseous lesion consistent with LSMFT with focal cystic change (Figure 2C,E). The patient was instructed to avoid weight‐bearing on the right lower limb for 3 months and to undertake knee range‐of‐motion exercises.

**FIGURE 3:**
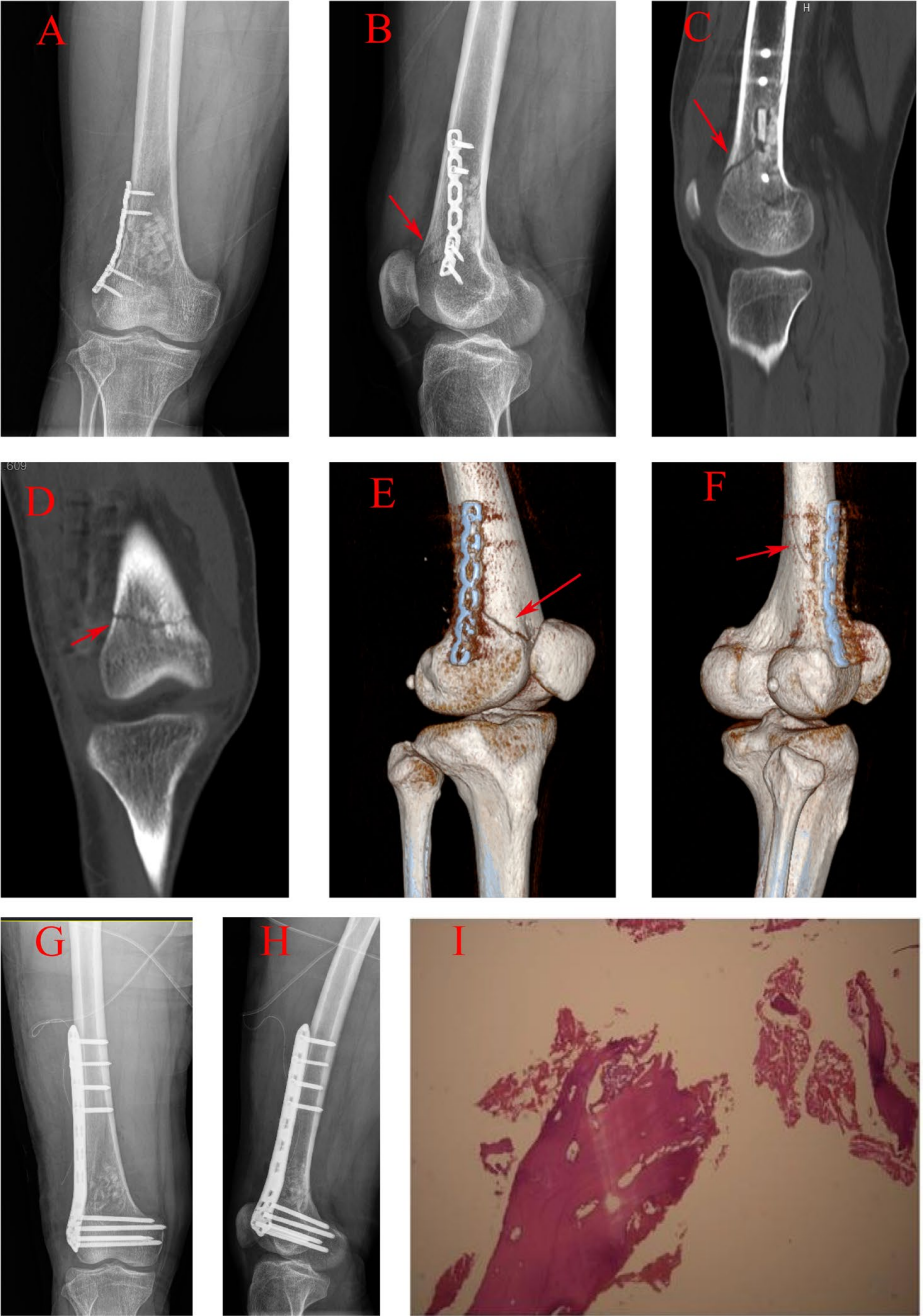
Presentation and management of the pathological fracture. (A, B) Radiographs 25 days after the initial surgery showing an oblique fracture at the previous resection site (red arrows). (C–F) Computed tomography and three‐dimensional reconstructions confirming the fracture around the lesion, with bone graft substitute present and no hardware failure (red arrows). (G–H) Radiographs after revision debridement, autologous iliac crest bone grafting, and fixation with a 7‐hole distal femoral plate. (I) Hematoxylin and eosin‐stained section showing no tumor recurrence or infection (original magnification ×4).

At fracture presentation, radiographs showed an oblique fracture at the previous resection site with approximately 2 mm of medial displacement of the distal fragment (Figure 3A,B). Computed tomography with three‐dimensional reconstruction confirmed a pathological fracture involving the anterior and posterior cortices around the lesion, with bone graft substitute within the resection cavity (Figure 3C–F). The LCP and locking screws showed no breakage, loosening, or other evidence of hardware failure. Tumor recurrence, infection, metabolic bone disease, and hardware failure were considered as potential contributors; imaging and normal laboratory results did not support these diagnoses.

After multidisciplinary discussion by the orthopedic oncology and trauma teams, revision surgery was performed on April 30, 2025, under subarachnoid anesthesia with the patient supine. Through the previous lateral incision, the fracture site was exposed and the prior bone graft substitute was debrided from the residual defect. The fracture was reduced with reduction forceps and temporarily stabilized with Kirschner wires; fluoroscopy confirmed anatomical reduction. Autologous cancellous bone was harvested from the right iliac crest and placed in the defect. A 7‐hole distal femoral plate (DFP; Mindray, China) was applied along the lateral cortex with screws proximal and distal to the fracture (Figure 3G,H). After debridement, the 1.8 cm × 1.5 cm × 1.5 cm defect was filled with the autologous graft, and the wound was closed in layers over a suction drain. Histopathological examination of the debrided tissue showed no tumor recurrence or infection (Figure 3I). Operative time was 90 min and estimated blood loss was 50 mL.

Postoperatively, intravenous cefazolin (1 g every 12 h) was administered for 48 h and parecoxib was used for analgesia. The drain was removed on postoperative day 2 after a total output of 80 mL. Rehabilitation began on postoperative day 3 with isometric quadriceps exercises and passive knee range‐of‐motion training, progressing from 0° to 90° over 2 weeks. The patient was discharged on postoperative day 5 using crutches with partial weight‐bearing (20% of body weight) for 6 weeks, followed by radiograph‐guided progression. Sutures were removed at 2 weeks; the wound healed without redness, drainage, or dehiscence.

## 4 Outcome and Follow‐Up

**FIGURE 4:**
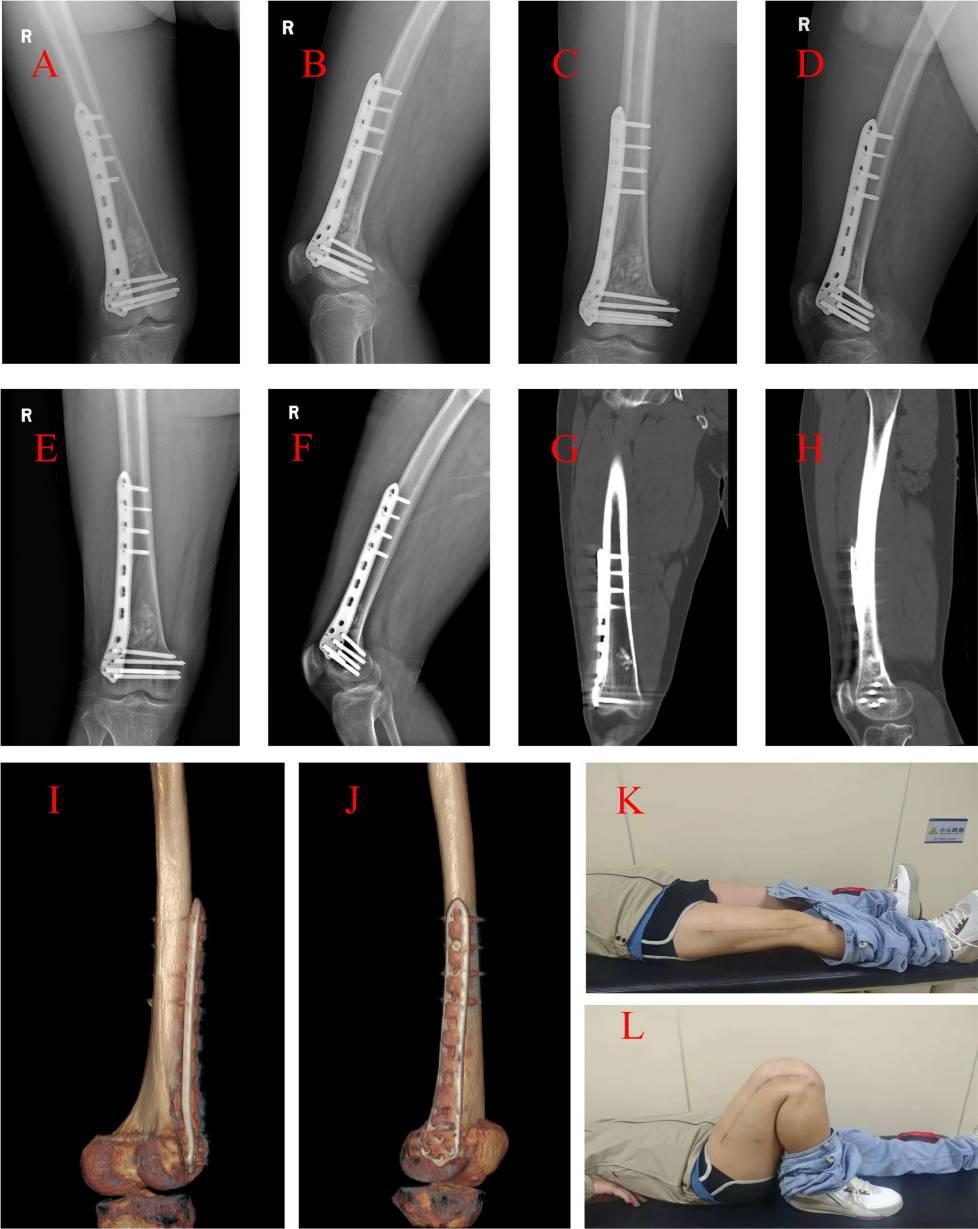
Postoperative follow‐up and functional recovery. (A,B) Radiographs at 1 month. (C,D) Radiographs at 3 months showing early callus formation without fixation loosening. (E–J) Radiographs and computed tomography images at 6 months confirming complete union and integration of the autologous bone graft. (K,L) Clinical photographs showing knee range of motion from 10° to 140°.

Follow‐up evaluations were performed at 1, 3, and 6 months. At 3 months, radiographs showed early callus formation without fracture displacement or loosening of the fixation (Figure 4A–D), and the patient progressed to full weight‐bearing. At 6 months, he had resumed all daily activities without limitation. Radiographs and computed tomography confirmed complete fracture union and integration of the autologous bone graft (Figure 4E–J). He reported no knee pain, achieved a knee range of motion of 10°–140° (Figure 4K,L), and had a Lysholm Knee Score of 98/100, indicating excellent function.

## 5 Discussion

LSMFT, first described by Ragsdale and Sweet in 1986 [1], was subsequently characterized by Kransdorf and colleagues as having a marked predilection for the intertrochanteric region of the proximal femur [8]. Subsequent series have confirmed that predilection: a 2016 narrative review indicated a “very strong predilection for the proximal femur” [3], an epidemiologic review reported that 85% of LSMFTs occur in the femur with 91% of these in the intertrochanteric region [9], and a 2025 systematic synthesis of 241 previously published cases confirmed the rarity of locations outside the proximal femur [7]. Although uncommon, LSMFT has been documented at atypical sites, including the distal femur [1], cranial vault [5], and other locations [10], as well as in a 9‐case series that included a distal‐femur lesion [11]. The case we present is therefore consistent with the broader experience that the distal‐femoral metaphysis is a rare but documented location for this entity.

The right distal femur is one of the highest‐stress regions of the lower extremity, particularly in the subchondral metaphyseal zone adjacent to the knee, where cortical thinning combined with a residual lesion‐related cavity can substantially reduce local bone strength [12]. In benign and low‐grade distal‐femur lesions, curettage with bone grafting alone does not always provide adequate mechanical stability, and prophylactic internal fixation is frequently warranted [13, 14]. Mirels‐type scoring can guide such decisions [15]. In our patient, the lesion measured 1.4 cm × 2.8 cm × 3.2 cm at the distal metaphysis (Figure 1F–H), which, although not exceptionally large, corresponded to a high‐stress periarticular environment. While the initial surgery combined curettage, artificial bone grafting, and 6‐hole LCP augmentation (Figure 2A,B), the fracture at 25 days postoperatively after a minor fall indicates that the construct had not yet matured enough to resist physiologic loads. Radiographic–histologic data show that the early phase of graft remodeling (within 8–12 weeks postoperatively) is dominated by osteoclastic resorption that temporarily weakens the construct before new bone deposition provides adequate strength [6, 16, 17], placing the patient in a “vulnerable window” of graft biology in which mechanical demand can transiently exceed biological reconstruction.

The principles of revision surgery for a pathological fracture through a previous resection cavity involve mechanically stabilizing the fracture and biologically reconstructing the residual bone defect. We therefore replaced the previous construct with a 7‐hole distal femoral plate and used autologous cancellous bone harvested from the right iliac crest after debriding the prior Rebone graft. The DFP was selected because it provides a dedicated distal‐femoral anatomical contour with multiple distal screw options, optimizing anchorage in the periarticular segment of the right distal femur, and offers a longer working length than the previous 6‐hole LCP, which is a recognized predictor of successful revision in distal‐femur nonunions [18, 19]. For biological reconstruction, autologous iliac‐crest bone graft was selected because of its osteoconductive, osteoinductive, and osteogenic properties [20]; the documented harvest‐site morbidity [19] did not occur in our patient. The revision procedure lasted 90 min with an estimated blood loss of 50 mL, and postoperative cefazolin was administered for 48 h. At the 6‐month follow‐up, X‐ray and CT confirmed complete fracture union with full integration of the autologous bone graft; knee ROM was 10°–140° and Lysholm score 98/100, consistent with excellent function. These clinical outcomes support the efficacy of secondary fixation combined with autologous bone grafting in managing post‐curettage pathological fractures of the distal femur [21].

To our knowledge, an early (< 30‐day) post‐curettage pathological fracture at the distal femur in LSMFT has rarely, if ever, been explicitly reported. Beytemür and colleagues [4] described a proximal‐femur LSMFT treated with curettage, grafting, and a 95° dynamic condylar screw‐plate; no post‐curettage fracture was observed at the 16‐month follow‐up. Zhang and colleagues [1] reported a distal‐femur LSMFT in a 55‐year‐old female treated with thorough curettage and bone grafting; similarly, no post‐curettage fracture occurred at the 6‐month follow‐up. Within the broader LSMFT literature, a secondary pathological fracture at a previous resection site is therefore a rare but clinically important complication [3, 7, 8]. Atypical‐site cases, such as the cranial‐vault lesion reported by Ploof and colleagues [5] and the unusual periarticular case reported by Barnds and colleagues [10], further illustrate that LSMFT can occur beyond its predominant intertrochanteric location and that secondary reconstruction strategies must be tailored to local anatomy. For the distal femur specifically, Holzman and colleagues [18] and Ebraheim and colleagues [19] have described revision strategies for distal‐femur nonunions following initial lateral locking‐plate fixation—including the addition of a medial locking plate and the use of iliac‐crest autograft—that yielded high union rates in selected cases. Our case parallels those reports: in a 38‐year‐old male with a right distal femur LSMFT, revision fixation with a longer distal‐femoral locking plate (7‐hole DFP) and autologous right iliac‐crest bone graft, after debridement of the previous Rebone calcium‐phosphate graft, yielded complete fracture union and excellent knee function (Lysholm 98/100) at the 1‐, 3‐, and 6‐month follow‐ups.

This case report has several limitations. First, it is a single‐case study, and the findings may not be generalizable to all patients. Second, long‐term data (≥ 5 years) on outcomes are lacking, and further studies with longer follow‐up are needed to assess the durability of treatment. Third, biomechanical testing of the construct (e.g., finite element analysis of the residual defect before and after the revised DFP construct, ideally using patient‐specific Quantitative CT data) was not performed, and prospective biomechanical evaluation of distal‐femur reconstruction strategies for LSMFT‐related defects would be valuable.

In conclusion, pathological fractures after primary resection of LSMFT are rare but manageable. Thorough debridement, revision autologous bone grafting, and stable internal fixation can achieve fracture union and excellent functional recovery. Long‐term follow‐up remains important to confirm graft integration and identify late recurrence or other complications.

## Author Contributions

**Yingsong Yuan:** conceptualization, data curation, formal analysis, investigation, methodology, visualization, writing – original draft. **Yang Gu:** conceptualization, data curation, formal analysis, investigation, methodology, supervision, writing – review and editing.

## Funding

This work was supported by Ningbo Clinical Research Center for Orthopedics, Sports Medicine & Rehabilitation (2024L004).

## Ethics Statement

This study was approved by the Institutional Review Board of Ningbo No. 6 Hospital.

## Consent

Written informed consent was obtained from the patient for publication of this case report and the accompanying images.

## Conflicts of Interest

The authors declare no conflicts of interest.

## Data Availability

The data that support the findings of this study are available from the corresponding author upon reasonable request.
